# Techniques for Improving Communication of Emotional Content in Text-Only Web-Based Therapeutic Communications: Systematic Review

**DOI:** 10.2196/mental.6707

**Published:** 2017-10-24

**Authors:** Christine Louise Paul, Martine Elizabeth Cox, Hannah Julie Small, Allison W Boyes, Lorna O'Brien, Shiho Karina Rose, Amanda L Baker, Frans A Henskens, Hannah Naomi Kirkwood, Della M Roach

**Affiliations:** ^1^ Priority Research Centre for Health Behaviour School of Medicine and Public Health University of Newcastle Callaghan Australia; ^2^ Hunter Medical Research Institute New Lambton Australia; ^3^ Hunter Cancer Research Alliance Waratah Australia; ^4^ Cancer Council New South Wales Woolloomooloo Australia; ^5^ School of Medicine and Public Health University of Newcastle Callaghan Australia; ^6^ Distributed Computing Research Group, School of Electronic Engineering and Computer Science, University of Newcastle Callaghan Australia

**Keywords:** nonverbal communication, Internet, computer-assisted therapy

## Abstract

**Background:**

Web-based typed exchanges are increasingly used by professionals to provide emotional support to patients. Although some empirical evidence exists to suggest that various strategies may be used to convey emotion during Web-based text communication, there has been no critical review of these data in patients with chronic conditions.

**Objectives:**

The objective of this review was to identify the techniques used to convey emotion in written or typed Web-based communication and assess the empirical evidence regarding impact on communication and psychological outcomes.

**Methods:**

An electronic search of databases, including MEDLINE, CINAHL, PsycINFO, EMBASE, and the Cochrane Library was conducted to identify literature published from 1990 to 2016. Searches were also conducted using Google Scholar, manual searching of reference lists of identified papers and manual searching of tables of contents for selected relevant journals. Data extraction and coding were completed by 2 reviewers (10.00% [573/5731] of screened papers, at abstract/title screening stage; 10.0% of screened [69/694] papers, at full-text screening stage). Publications were assessed against the eligibility criteria and excluded if they were duplicates, were not published in English, were published before 1990, referenced animal or nonhuman subjects, did not describe original research, were not journal papers, or did not empirically test the effect of one or more nonverbal communication techniques (for eg, smileys, emoticons, emotional bracketing, voice accentuation, trailers [ellipsis], and pseudowords) as part of Web-based or typed communication on communication-related variables, including message interpretation, social presence, the nature of the interaction (eg, therapeutic alliance), patient perceptions of the interaction (eg, participant satisfaction), or psychological outcomes, including depression, anxiety, and distress.

**Results:**

A total of 6902 unique publications were identified. Of these, six publications met the eligibility criteria and were included in a narrative synthesis. All six studies addressed the effect of smileys or emoticons on participant responses, message interpretation, or social presence of the writer. None of these studies specifically targeted chronic conditions. It was found that emoticons were more effective in influencing the emotional impact of a message than no cue and that smileys and emoticons were able to convey a limited amount of emotion. No studies addressed other techniques for conveying emotion in written communication. No studies addressed the effects of any techniques on the nature of the interaction (eg, therapeutic alliance), patient perceptions of the interaction (eg, participant satisfaction), or psychological outcomes (depression, anxiety, or distress).

**Conclusions:**

There is a need for greater empirical attention to the effects of the various proposed techniques for conveying emotion in Web-based typed communications to inform health service providers regarding best-practice communication skills in this setting.

## Introduction

The majority of people with chronic disease may experience some level of psychological distress in their lifetime [1,2]. Compared with the general population, depression is more prevalent in all disease groups, and anxiety is more prevalent in people living with chronic diseases such as cardiovascular disease, stroke, and cancer [1,2]. The heavy symptom burden associated with chronic conditions often translates into increased physical impairment and reduced functional capacity [3,4].

Various forms of psychological support have been found to improve physical and psychological outcomes in patients with chronic conditions [2,5,6]. Approaches for providing psychological support include face-to-face interventions such as clinician-led support groups, one-on-one, and group counseling; written interventions such as information booklets and therapy workbooks; telephone-based interventions, including telephone counseling; and comparatively newer Web-based modalities of support, including Web counseling (eg, synchronous Web-based chat or asynchronous email exchange). Web-based or computer-mediated communication can produce similar outcomes to face-to-face approaches for alleviating psychological burden in patients with chronic conditions and psychiatric and somatic disorders [5,7,8]. A 2014 meta-analysis by van Beugen et al [5] demonstrated that Internet-delivered therapy is comparable in efficacy with group-based face-to-face therapy. Similarly, a 2013 review of effectiveness studies by Andersson et al [7,8] found Internet-based therapy to be of equivalent effectiveness to face-to-face therapy in a group setting. Moreover, a 2014 systematic review and meta-analysis by Andersson et al [7] determined that Internet-based therapy produced overall effects equivalent to face-to-face therapy. Textual Web-based interactions can develop an effective alliance between a therapist and client [9] and may be helpful in relationship-building for those with social anxiety [10]. The systematic review and meta-analysis by Andersson et al [7] demonstrated equivalent effects of Internet-delivered therapy and face-to-face therapy for social anxiety disorder in favor of the Internet-based modality. Furthermore, Lundy and Drouin [10] found that those experiencing social anxiety reported greater feelings of interpersonal connectedness following engagement with a synchronous instant messaging platform, demonstrating the ability of text-only mediums of communication to contribute to psychological well-being. Accordingly, Web-based forms of communication are increasingly being adopted by both government and nongovernment health services as an important mode for providing social and emotional support. Therefore, it is important that the communication parameters required for optimal effectiveness are well understood.

The advantages of Web-based or computer-mediated communication over face-to-face or telephone-based methods of providing psychological support include fewer limitations related to geographical location, staffing, resources, and timing of service provision, as well as anonymity for the patient [11]. It has been proposed that text-based communication techniques are construed by the Web patient in the same way that nonverbal communication methods are by the face-to-face client and that these Web-based communication methods can serve the same purpose in supporting positive interaction as in face-to-face situations [12]. However, a number of theories, such as the cuelessness model [13] and social information processing theory [14], suggest that a lack of visual and auditory cues presents challenges to the patient and provider in their attempts to convey emotional content via Web-based communication methods. The lack of nonverbal communication in Web-based exchanges is thought to limit message interpretation, create psychological distance, and reduce rapport [15]. Online communication techniques that have been suggested to counteract this lack of visual and auditory cues include smileys (stylized graphic images of facial expressions used to convey emotion or feeling), emoticons (use of American Standard Code for Information Interchange punctuation marks, numbers, and letters to create pseudofacial expressions to convey the writer’s emotion or intended tone), emotional bracketing (using words or text to describe emotions in brackets), voice accentuation (bolding, underlining, or italicizing words or text), trailers (ellipsis), and pseudowords (a fake word constructed from a string of letters).

Whereas telehealth has been highlighted as having potential relevance to this review, it is not considered to be directly applicable as it is outside the scope of the research question. Despite some definitions of telehealth, including broad concepts such as text-based communication between a health care professional and a patient without the presence of videoconferencing contact, the literature largely focuses on patient outcomes rather than conveyance of emotion. Furthermore, the literature regarding health care professional and patient relationships with respect to the provision of telehealth services seems to relate to emotional communication transmitted by tone of voice, posture, gaze, and eye contact, which is irrelevant to the explicit focus of this review (communication methods in Web-based text-only interactions).

Although some empirical evidence exists to suggest that Web-based or typed communication techniques have the ability to convey emotion [16] and are used in response to a need to convey emotion in text-only communications [17,18], there is no systematic review of the evidence to guide practitioners regarding the use of the available strategies. This review attempts to assess the effects of text-only (written or typed) Web-based communication on emotional experience. It also explores the implications for therapeutic communication with the aim of improving psychological outcomes for people with chronic disease.

The review aims to critically examine original research to identify experimental studies regarding Web-based communication methods to describe the following:

1. The number of empirical studies of techniques used to convey emotion in written or typed Web-based communication (eg, email counseling or Web counseling).
2. The effect of the identified techniques on communication-related variables, including message interpretation, social presence, the nature of the interaction (eg, therapeutic alliance), and patient perceptions of the interaction (eg, participant satisfaction).
3. The impact of the identified techniques on patient psychological outcomes, including depression, anxiety, and distress.

## Methods

### Design

An electronic search of databases, including MEDLINE, CINAHL, PsycINFO, EMBASE, and the Cochrane Library was conducted to identify literature published from 1990 to 2016. Searches were also conducted using Google Scholar, manual searching of reference lists of identified papers, and manual searching of tables of contents for selected relevant journals.

### Search Strategy

#### Development of Search Strategy

Two medical librarians provided expert guidance on the development of the review’s search strategy. The initial search strategy was developed and tested by the research team and forwarded to the first medical librarian for review regarding search terms and appropriate databases. There was significant overlap of search terms in the original and alternative search strategies, including *professional-patient relations*, *therapeutic alliance*, *computer*, *cyber*, email*, *web*, *online*, *internet,* and instant mess*. Strategies suggested by the first medical librarian, which were integrated into the final search strategy, included implementation of the adj5 function to search for 5 words or fewer apart from *counsel* and *therap*, explosion of certain medical subject headings or MeSH terms (*emotion*), and confirmation of the decision to exclude Sociological Abstracts from the list of databases searched because of the largely irrelevant results it yielded. The second medical librarian reviewed the final search strategy as a quality assurance exercise and ran an alternative approach to the search. The results of the alternative search strategy were exported into an EndNote X7.3.1 database and compared with the results of the original search strategy. This comparison demonstrated that the alternative search strategy did not yield any additional relevant content. The search terms utilized for each database is provided in Table 1.

#### Final Search Strategy

The search was limited to papers published from January 1, 1990 to December 31, 2016. The year 1990 was chosen as the starting point of the literature search strategy period as the availability of Web-based technology was greatly accelerated during the 1990s.

**Table 1:** Search terms utilized for each database.

Database	Search terms
MEDLINE	[Emotions OR satisfaction (kw) OR psychosocial (kw) OR Depressive Disorder OR distress (kw) OR Depression OR Anxiety OR Anxiety Disorders OR Professional-Patient Relations OR therapeutic alliance (kw)] AND [Electronic Mail OR e-therapy (kw) OR helpline (kw) OR ((computer or cyber or email* or web or online or internet or instant mess*) adj5 counsel*) (kw) OR ((cyber or email* or web or online or internet or instant mess*) adj5 therap*) (kw)].
EMBASE	[Emotion OR patient satisfaction OR psychosocial (kw) OR depression OR anxiety OR distress (kw) OR doctor patient relation OR therapeutic alliance (kw) OR professional-patient relations (kw)] AND [e-therapy (kw) OR helpline (kw) OR e-mail OR ((computer or cyber or email* or web or online or internet or instant mess*) adj5 counsel*) (kw) OR ((cyber or email* or web or online or internet or instant mess*) adj5 therap*) (kw)].
PsycINFO	[Emotions OR client satisfaction OR psychosocial factors OR depression (kw) OR depression (emotion) OR anxiety disorders OR anxiety OR distress OR therapeutic alliance] AND [Computer mediated communication OR electronic mail (kw) OR email (kw) OR electronic communication OR e-therapy (kw) OR online therapy OR ((computer or cyber or email* or web or online or internet or instant mess*) adj5 counsel*) (kw) OR ((cyber or email* or web or online or internet or instant mess*) adj5 therap*) (kw) OR helpline (kw)].
CINAHL	[Emotions OR Satisfaction OR Psychosocial OR Depressive Disorder OR Depression OR Distress OR Anxiety OR Anxiety Disorders OR Professional-Patient Relations OR Therapeutic Alliance] AND [Helpline OR Electronic Mail OR e-mail OR e-therapy OR Cybercounsel* OR Cybertherap* OR Online N5 counsel* OR Online N5 therap* OR Cyber N5 counsel* OR Cyber N5 therap* OR Computer N5 counsel* OR Email N5 counsel* OR Email N5 therap* OR Web N5 therap* OR Web N5 counsel* OR Internet N5 counsel* OR Internet N5 therap* OR Instant mess* N5 counsel*].

### Study Inclusion and Exclusion

The inclusion criteria were developed to isolate studies that identified the effects of communication techniques used in typewritten Web-based communication. Variables that were only relevant to audiovisual communication were not sought. All eligible abstracts were examined for relevance following removal of duplicates. The full-text papers of potentially relevant original research studies were obtained and examined. A coding template was used to extract relevant data from the included publications. Publications were excluded if they were duplicates, were not published in English, were published before 1990, referenced animal or nonhuman subjects, did not describe original research, were not journal papers, or did not empirically test the effect of one or more nonverbal communication techniques (for eg, smileys, emoticons, emotional bracketing, voice accentuation, trailers [ellipsis], and pseudowords) in adults as part of Web-based, Web-based or typed communication on communication-related variables, including, for example, message interpretation, social presence, the nature of the interaction (such as therapeutic alliance), patient perceptions of the interaction (such as participant satisfaction), or psychological outcomes, including depression, anxiety, and distress.

Article abstracts were initially assessed against the eligibility criteria by MC and excluded if the study did not meet the inclusion criteria. A random subsample (10.0%) of included studies were categorized by 2 other authors (CP and HS) and compared with the coding of MC. An initial agreement of 83.0% was achieved, with any discrepancies resolved via discussion and recoded as necessary.

#### Classification and Inclusion of Publications by Focus Area

All publications which met the eligibility criteria were coded by CP, and 10.0% were independently coded by another author (AWB, agreement=91.0%) to determine the papers that focused on the following:

Techniques used to convey emotion in written or typed Web-based communication: publications that reported techniques used (eg, smileys, emoticons, emotional bracketing, voice accentuation, trailers [ellipsis], and pseudowords); communication modality (eg, Web-based chat and email); format of communication delivery (eg, asynchronous communication and synchronous communication); and one of the following:

1. The effect of the identified techniques on message interpretation and social presence: publications that reported effect on mood or perception of writer’s commitment, perceived extraversion or sociality of writer, and perception of message (perceived emotion, attitude reception, or attention perception).
2. The effect of the identified techniques on the nature of the interaction: publications that reported effect on therapeutic alliance or working alliance.
3. The effect of the identified techniques on patient perceptions of the interaction: publications that reported effect on participant satisfaction.
4. The impact of the identified techniques on patient psychological outcomes: publications that reported effect on psychological outcomes, including depression, anxiety, and distress.

#### Classification and Inclusion of Publications by Design

The full-text of intervention studies was then assessed independently by 2 reviewers (MC and CP) regarding whether those studies met the minimum design criteria for classification as any one of a randomized controlled trial (RCT), a non-RCT, a controlled before and after study, or an interrupted time series study using the Cochrane Effective Practice and Organization of Care risk of bias criteria. There was perfect agreement (100.0%) between the 2 reviewers.

### Data Extraction and Coding of Included Studies

To assess intervention effectiveness, data on each of the following variables were extracted by 2 authors (CP and DR) using an extraction coding table: author and year of publication, study setting; study design, sample characteristics (sample size, gender, and age), assessment based on inclusion and exclusion criteria, outcome measures, and study findings. An agreement of 96% was achieved (a single error was identified and corrected).

## Results

**Figure 1:**
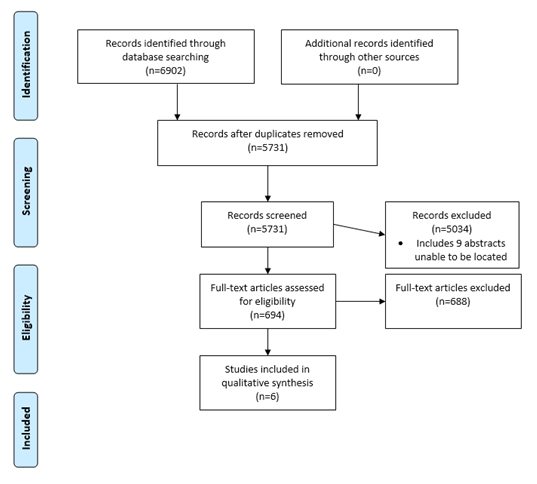
Preferred Reporting Items for Systematic Reviews and Meta-Analyses: The PRISMA Statement – flow chart of search strategy and paper selection.

A total of 6902 publications were identified using the search strategy (see Figure 1 for PRISMA flow diagram) [19]. After duplicates were removed, 5731 publications were assessed against the eligibility criteria. The abstracts of nine individual publications were unable to be located. A total of six publications met the eligibility criteria and were included in the review. The studies excluded at the full-text stage either (1) described but did not test the techniques of interest or (2) described the effects of computer-mediated interventions but did not address the effect of any communication technique used within the intervention (over 90%). That is, whereas there are many studies that explore Web-based communication, the effects of the communication techniques themselves have not generally been isolated as part of the study design. None of the included studies specifically targeted chronic conditions. The six included studies are described in full in Multimedia Appendix 1. The study data were not suitable for meta-analysis, as there was no commonality of measurement.

All of the six studies addressed the effect of smileys or emoticons on message response, message interpretation, or social presence of the writer. No studies addressed other techniques for conveying emotion in text-only typed communication. No studies addressed the effects of any text-only typed communication techniques on the nature of the interaction (eg, therapeutic alliance), patient perceptions of the interaction (eg, participant satisfaction), or psychological outcomes (depression, anxiety, or distress).

Four of the included studies found that the use of a smiley or emoticon is more effective than no cue in influencing the perceived emotion of an interaction [14,20-22]. Walther and D'Addario [16] established that the use of smileys or wink emoticons is unable to decrease the negative tone of a negatively worded message. In addition, Walter and D'Addario [16] observed that the inclusion of any negative element (words or cue) caused the reader to experience a negative change in interpretation of the Web-based interaction. Thompson et al [22] demonstrated higher levels of arousal and increased smiling and reduced frowning when an emoticon is present, supporting the argument that use of an emoticon is more effective at eliciting a positive response in the viewer when compared with no cue. Furthermore, the findings of Thompson et al [22] support the notion that emoticons augment the emotional impact of a message by demonstrating increased smiling as a result of phrases conveying praise when an emoticon is present, compared with a praising statement when an emoticon is absent.

Walter and D'Addario [16] observed that words elicit a stronger response than emoticons, whereas a later study by Comesana et al [23] found the opposite effect—that smileys cause greater brain activity than words.

Lo [20], Ganster et al [21], and Wall et al [24] studied the effect of emoticons and smileys on perception and evaluation of the writer’s qualities (including perceived emotion, attitude, perception, commitment, extraversion, sociality, agreeableness, conscientiousness, and openness). Lo [20] observed the ability of emoticon use to affect perceived attitude (like vs dislike) and perceived attention of the writer in the interaction, whereas Ganster et al [21], in a study of cue valence effects on perceptions of the writer’s personality, found that positive cue use (compared with negative cue use or cue absence) by the author denotes a greater perception of writer extraversion with no effect on their perceived commitment or sociality. Furthermore, Ganster et al [21] observed a stronger effect of smileys on the perceived commitment of the writer when compared with emoticons; however no effect on perceived extraversion or sociality of the author was found. Wall et al [24] provided more recent evidence for the effect on perception and evaluation of the writer’s traits by demonstrating positive correlations between the writer’s use of *happy* emoticons and the observer’s assessment of qualities of agreeableness, conscientiousness, and openness.

## Discussion

### Principal Findings

Very few experimental studies have explored the effects of typed strategies to assist conveyance of emotion in text-only forms of communication. Although there are many studies of the efficacy of computer-mediated psychotherapeutic interventions, there were no studies exploring the impact of communication techniques for conveying emotion on the nature, perception, or outcomes of such interactions. The studies excluded at the full-text stage either described computer-mediated interventions without addressing the effect of communication techniques or were observational (described the use of rather than tested the effects of such techniques). No studies regarding communication techniques other than smileys and emoticons were identified. These two aspects of computer-mediated communication highlight important gaps in the evidence base regarding the delivery of supportive or therapeutic typed Web-based communication.

The six experimental studies identified indicate that smileys and emoticons do strengthen the perceived emotion in text-only messages. The work of Comesana et al [23] suggests that emoticons evoke a rapid and automatic emotional response even before the receiver is aware of what he or she has seen. Therefore, these techniques can be used to ameliorate the psychological distance and lack of social presence, which is theorized to occur in text-only communication as a result of the absence of social cues and retardation of impression formation [13,14]. Walter and D'Addario [16] and Thompson et al [22] found that the emotional tone of a message can be positively influenced through use of a smile or wink emoticon, suggesting the advantageous use of these emoticons in the therapeutic environment. Use of an emoticon is more effective than not using an emoticon in affecting the perceived emotion (positive or negative) of the interaction, also giving strength to the usefulness of these communication tools in Web-based interactions [20,22]. Similar to the findings of Lo [20], the study by Ganster et al [21] found that use of an emoticon or smiley conveys more positive (smile) or negative (frown) emotion when compared with the absence of a cue. Ganster et al [21] also found that smileys have a stronger effect than emoticons on the expression of the perceived commitment of the conversation moderator, which could be considered an important influence on working alliance in the therapeutic setting. Comesana et al [23] found that more brain activity on electroencephalogram was elicited by smileys over words and negative smiley-and-word pairings, again strengthening the argument for the use of smileys, while also encouraging sensitivity with respect to use of valenced text-only communication tools in Web-based interactions.

There appear to be some caveats on what smileys and emoticons can achieve as techniques for conveying emotion: positively valenced emoticons cannot counteract a negatively worded message, and there appears to be a particular sensitivity to negative smileys such that they may need to be utilized sparingly [23]. Furthermore, these studies have only explored the expression of a very limited range of emotions, that is, happy or sad smileys or emoticons. The expression of, and response to, more complex emotions via smileys or emoticons is likely to be difficult, given that one nonexperimental study of the understanding of smileys found poor recognition of smileys or emoticons other than those relating to a limited range of emotions [25].

Current literature indicates that therapeutic interventions delivered Web-based can be as successful as the face-to-face versions [9,10], suggesting that computer-mediated communication can achieve a strong therapeutic alliance and working relationship. However, a lack of understanding of the most appropriate ways in which to optimize this relationship (including the communication of emotion) may be a limiting factor. The need for study of techniques for expressing more complex emotions in text-only Web-based exchanges is crucial. The Walther and D'Addario [16] study suggests that other techniques such as emotional bracketing may be even more powerful than smileys, given that positively or negatively worded statements were found to have a stronger effect than emoticons on perception. There is a need for empirical examination of the use of the emotional bracketing method in clinical settings to provide therapists with practical guidance on how to effectively use this technique in Web-based therapeutic environments. Further research should particularly focus on the effect of smileys, emoticons, and other communication techniques (including emotional bracketing) on factors including therapeutic alliance and participant satisfaction. Therapeutic alliance is a dependable predictor of a favorable clinical outcome [26]. Furthermore, future research has the potential to provide novel evidence on the impact of the identified techniques on patient psychological outcomes, including depression, anxiety, and emotional distress. There is compelling evidence to suggest that Web-based therapy is effective in improving clinical outcomes in patients with depression, anxiety, and emotional distress among chronic disease and cancer populations [27]; however, further research on the effects of Web-based communication techniques in these populations is lacking and requires prompt attention.

### Limitations

It is important to acknowledge that the identified studies have been conducted with young and healthy nonclinical populations, rather than patient populations. This significantly limits the ability to generalize the findings to older people or to patient populations. For older people and patient populations, there is perhaps a greater need for sensitivity in conveying emotion in a text-only setting [28,29]. There have been previous intervention studies whereby successful interventions conducted in older people and chronic disease populations have demonstrated a heightened sensitivity with regard to communication methods to be a crucial element in interactions with these cohorts [28,29].

### Conclusions

This review suggests that the use of a limited range of easily recognized smileys and emoticons could be cautiously encouraged in text-only therapeutic interactions for people in younger age groups. There is a need for further research on how to use communication techniques, including emoticons and smileys, effectively in text-only interactions across a diverse range of ages and to inform evidence-based practice of therapists working in the Web-based domain. Furthermore, there is a need for research focused on the communication of emotion in text-only Web-based interactions in a therapeutic clinical setting.

## Appendix

Multimedia Appendix 1 Data extracted from the four studies included in the review.

## Abbreviations

RCT: randomized controlled trial
