# Supplementary material for: Techniques for Improving Communication of Emotional Content in Text-Only Web-Based Therapeutic Communications: Systematic Review
Source: JMIR Ment Health. 2017 Oct 24;4(4):e46. doi: 10.2196/mental.6707 (PMC5676033; doi:10.2196/mental.6707)
Supplement: Multimedia Appendix 1 [file mental_v4i4e46_app1.pdf]

Author, year, and country	Sample recruitment method	Design	Comparison conditions	Outcomes	Findings
Walther and D'Addario, 2001 [16], United States	226 university students:<br>- 71.0% male (M), 29.0% female (F)<br>- Mean age 19 years<br><br>Class participation	Randomized 4x2 between-subjects design<br><br>View single email	Message recourse:<br>- Emoticon (smile, frown, wink, and no cue)<br><br>Positive or negative statement	- Message interpretation (confusing, serious, and ambiguous)<br>- Writer interpretation (attitude, happiness, and sincerity)<br>- Social influence (likelihood of taking course)<br>- Degree of emotion portrayed	- No gender effect<br>- Words have stronger effect than emoticons (course attitude *P*<.001; happiness *P*=.015; message ambiguity *P*=.012)<br>- Smile or wink emoticon makes message seem happier than no cue (*P*<.001)<br>- Smile or wink does not decrease negativity of negative message<br>- Any negative element (text or cue) causes negative “shift” in interpretation
Lo, 2008 [19], Taiwan	137 instant message users (no gender or age data collected)	Random assignment to one of 3 groups with 3 within-subjects conditions	3 types of social messages, each with emoticon (positive, negative, and none)	Writer’s perceived emotion, attitude, and attention	- Using an emoticon affects perceived emotion (positive or negative) compared with no emoticon (*P*<.001)<br>- Using an emoticon affects perceived attitude (like vs dislike) (*P*<.001)

					- Using an emoticon affects perceived attention (*P*<.001)
Ganster et al, 2012 [20], Germany	127 volunteers:<br>- 26.0% M, 74.0% F<br>- Mean age 24 years<br><br>Open online survey platform	Randomized 2x2 between-subjects + neutral condition<br><br>View chat dialogue between 2 others reexam	Discussion reupcoming exams plus<br>- Smiley versus emoticon<br>- Positive (smile) versus negative (frown) cue<br>- No cue	- Message interpretation (positive and humorous)<br>- Evaluation of writer of cue (commitment extraversion and sociality)<br>- Own mood	- Emoticon or smiley makes message seem more positive (smile) or negative (frown) compared with no cue (*P*<.001)<br>- Writer is perceived as more extroverted if using cue but no effect on commitment or sociality (*P*<.001)<br>- Rater has lower mood if sees frown cue (*P*<.05)<br>- Smileys have stronger effect than emoticons on perceived commitment only (*P*<.05)
Comesana et al, 2013 [22], Spain	18 university students<br><br>Exposure to stimuli	All participants received all stimuli in counterbalanced order	Subliminal presentation of<br>(1) pleasant smiley,<br>(2) unpleasant smiley,<br>(3) pleasant word,<br>(4) unpleasant word before presentation of	- Electroencephelo graphic (EEG) activity<br>- Reaction time (choice of whether target word was positive or negative)	- Smileys cause a larger EEG response than words (*P*<.01)<br>- Unpleasant smiley and unpleasant word had a larger EEG response (*P*<.05) than positive smiley + target word pairs,

			pleasant or unpleasant target words		incongruent smiley + target word pairs, and words-only pairs<br>- Smileys associated with shorter reaction times than words ($P<.05$)
Thompson et al, 2016 [21], United Kingdom	47 university students:<br>- English speaking<br>- 34.0% M, 66.0% F<br>- Mean age 24 years<br><br>Open online survey platform<br><br>Exposure to stimuli	Randomized 2 polarity (praise vs criticism) x 2 literality (literal vs ironic) x 2 emoticon (present vs absent) within-subject design<br><br>160 items, each with 8 conditional variants<br><br>All participants received all items in counterbalanced order (4 blocks with 40 items in each)	Presentation of items:<br>(1) Phrase featuring critical and ironic statement with emoticon<br>(2) Phrase featuring critical and ironic statement full stop<br>(3) Phrase featuring critical and literal statement with emoticon<br>(4) Phrase featuring critical and literal statement with full stop<br>(5) Phrase featuring praising and ironic statement with emoticon<br>(6) Phrase	- Electrodermal activity to measure arousal<br>- Facial electromyography to detect muscle movements indicative of emotional expressions	- Higher arousal when emoticon present ($P<.05$)<br>- Reduced frowning ($P<.01$) and increased smiling ($P<.05$) when emoticon present<br>- Reduced smiling for praising statement when emoticon absent ($P<.008$)<br>- Greater smiling for phrases conveying praise when emoticon present<br>- Emoticons have an important function in clarifying intent<br>- Emoticons assist with modulating emotional impact of a message and aiding comprehension

			featuring praising and ironic statement with full stop<br>(7) Phrase featuring praising and literal statement with emoticon<br>(8) Phrase featuring praising and literal statement with full stop		
Wall et al, 2016 [23], United Kingdom	7 undergraduate psychology university students:<br>- 2 male, 5 female	Naïve observers rated first impression of online chat of unacquainted target participants who had previously completed a counterbalanced self-report questionnaire on emoticon use, then participated in the online chat conversation with an unknown user	Observation of emoticon use in online chat discussion about everyday life:<br>(1) Presence or absence of "happy" emoticon	- Observers' impressions of personality judgments using modified *International Personality Item Pool* (5 traits: extraversion, agreeableness, conscientiousness, neuroticism, and openness)<br>- Assessment of accuracy of observers' impression—correlation between aggregate observer ratings and participant self-report	- Positive correlations found between targets' use of "happy" emoticons and observers' assessment of agreeableness (*P*<.01), conscientiousness (*P*<.05), and openness (*P*<.05)

				- Correlation between emoticon use and personality traits measured	
